# Gene Families, Structural and Functional Contexts Shape Protein Thermostability and Paralog Divergence in *Arabidopsis*

**DOI:** 10.3390/plants15172617

**Published:** 2026-08-27

**Authors:** Yanli Cheng, Wenqing Zhang, Feng Sun, Zhitao Hu, Zongbo Han, Junlong Zhou, Haining Dong, Weizhong Liu, Wenqing Shi

**Affiliations:** College of Life Sciences, Shanxi Normal University, Taiyuan 030031, China; yanlicheng@sxnu.edu.cn (Y.C.);

**Keywords:** *Arabidopsis thaliana*, thermal proteome, protein thermostability, paralog divergence, gene family

## Abstract

Protein family membership may constrain protein thermal stability, but the extent of this effect remains unclear. We reanalyzed a published thermal proteome dataset containing 3917 *Arabidopsis thaliana* proteins. Proteins within the same family showed significantly smaller pairwise T_m_ differences than size-matched random groups, and family membership explained 67.6% of total T_m_ variation. Although thermal stability was generally conserved within families, some paralogs displayed marked divergence beyond that expected from sequence similarity. This exceptional divergence was not associated with duplication mode or overall structural distance, whereas differences in disulfide-bond density showed the clearest structural association. Gene Ontology cellular-component divergence was also positively associated with thermal divergence. Overall, thermal stability is strongly organized at the gene-family level, while exceptional divergence among paralogs appears to arise through heterogeneous, pair-specific structural and functional changes.

## 1. Introduction

Temperature is a pervasive environmental variable that directly affects protein folding, conformational dynamics, molecular interactions, catalytic activity, and protein solubility [1,2,3]. This thermal stress challenge is particularly critical for plants, as sessile organisms that cannot evade rapid diurnal and seasonal temperature fluctuations [4]. Genetic and proteomic studies in *Arabidopsis thaliana* have established that molecular chaperones, including HSP101 and small heat-shock proteins, are indispensable for preventing irreversible protein aggregation, stabilizing translation-associated proteins, and facilitating plant recovery from heat stress [5,6,7,8]. At the individual-protein level, protein thermal behaviour is typically described by the apparent melting temperature (T_m_), which defines the threshold temperature at which a large proportion of soluble proteins undergo unfolding, aggregation, or depletion from the soluble cellular fraction [9]. Notably, the apparent T_m_ detected in biological samples is not solely determined by amino acid sequence or intrinsic protein folding free energy, but is also modulated by ligand binding, post-translational modification, protein-protein interactions, macromolecular-complex assembly, and intracellular physicochemical conditions [10]. The development of the cellular thermal shift assay (CETSA) and mass spectrometry-based thermal proteome profiling (TPP) enables high-throughput quantification of these context-dependent protein thermal properties across thousands of proteins, establishing apparent protein thermal stability as a robust systems-level molecular phenotype [1,9,11,12,13,14,15].

Subsequent developments in thermal proteomics have substantially broadened the biological interpretation of protein melting behavior. Changes in protein thermal stability have been utilized to identify ligand-protein and drug-target interactions, monitor membrane-protein binding, characterize metabolite-binding proteins, and investigate protein states in intact cells, tissues, and microorganisms [9,12,13,14,15,16,17,18,19,20,21,22]. Proteins within the same molecular complex frequently display coordinated thermal precipitation profiles, enabling thermal coaggregation to serve as a proxy for complex organization and remodelling [23]. Furthermore, proteome-wide thermal behaviour varies with cell cycle progression, metabolic status, and cellular perturbations [24,25]. ATP and other metabolites alter protein solubility and thermal stability via direct binding, as well as indirect effects on protein complexes and cellular organization [16,19]. Cross-species thermal profiling has further uncovered widespread evolutionary variation in proteome stability and demonstrated that sequence conservation, protein architecture, complex membership, and cellular environment jointly shape protein melting behaviour [17,20,22,26]. Collectively, these findings highlight that apparent T_m_ should be interpreted as an integrated biochemical phenotype, rather than a direct, isolated readout of equilibrium thermodynamic stability.

Despite the importance of thermal stability for sessile organisms, plant thermal proteomes have received substantially less attention than those of mammalian cells, microorganisms, or cultured cell lines. The first genome-scale thermal profiling study in *Arabidopsis* produced highly replicated melting curves for more than 1700 proteins and identified associations between apparent T_m_ and protein molecular weight, charged and polar amino-acid composition, secondary structure, and hydrophobic surface properties [27]. More recently, thermal profiling under native-like *Arabidopsis* cellular conditions quantified thousands of proteins and uncovered thermosensitive and thermoresilient protein networks, proteostasis-related vulnerabilities, coordinated melting of interacting proteins, and variation in the thermal behaviour of paralogous proteins [10,28]. While these studies laid a foundation for plant thermal proteomics and demonstrated that protein melting profiles encode information about both cellular organization and protein evolution, they have largely treated proteins as independent entities, components of discrete complexes, or isolated paralog pairs. Consequently, the extent to which proteome-wide thermal stability is hierarchically organized by gene-family membership remains insufficiently understood.

Gene families originate mainly from gene and genome duplications, which constitute a key source of evolutionary innovation prevalent in flowering plants. Classical models propose that duplicated genes can undergo loss, retain redundant ancestral functions, partition ancestral functions via subfunctionalization, or evolve novel functions via neofunctionalization [29,30]. The *Arabidopsis* genome carries extensive signatures of ancient whole-genome duplication, segmental duplication, and local gene-duplication events, which collectively generated a highly dynamic genome populated by numerous multigene families [31,32,33,34,35]. Following duplication, retained gene copies can diverge in coding sequence, gene architecture, expression abundance, tissue specificity, stress responsiveness, interaction partners, biochemical activity, and subcellular localization [34,35,36,37,38,39,40,41,42,43,44,45,46,47,48]. Such divergence is non-random. Whole-genome duplication tends to preserve dosage-sensitive genes, pathways, and molecular complexes, whereas tandem, proximal, transposed and dispersed duplications expose individual copies to distinct regulatory and selective environments [35,36,37,40,42,43,44,45]. In *Arabidopsis*, tandemly expanded gene families are frequently linked to biotic and abiotic stress responses, while genes retained after whole-genome duplication are enriched in regulatory and dosage-sensitive functions [35,36,40]. Duplicate genes may also partition ancestral splice isoforms or acquire distinct intracellular localizations, creating additional routes for functionally differentiating initially redundant proteins [46,47,48].

Compared with divergence at the sequence, expression, and regulatory levels, the biophysical consequences of gene duplication remain poorly characterized. Protein thermal stability provides a quantitative protein-level phenotype to assess conservation and diversification among paralogs. Closely related paralogs typically maintain similar folds and physicochemical properties, leading to conserved thermal stability shortly after duplication. Nevertheless, sequence substitutions, insertions or deletions, altered oligomerization, shifted ligand specificity, rewired interactors or changed subcellular distribution can drive divergence in thermal behavior. Importantly, raw pairwise T_m_ differences cannot distinguish thermal divergence expected from sequence evolution from divergence that is unusually large or small given a fixed level of sequence similarity. A sequence-adjusted analytical framework is therefore required to pinpoint paralog pairs whose thermal divergence exceeds or falls below sequence-based predictions.

Protein architecture offers one plausible mechanistic basis for such divergence. Comparisons between mesophilic and thermophilic proteins link thermal stability to a broad set of structural traits: compactness, solvent accessibility, hydrophobic packing, salt-bridge networks, electrostatic interactions, hydrogen bonding, secondary-structure composition, loop content and oligomeric organization [49,50,51,52]. However, the impact of these features depends strongly on protein family and structural context. Features that stabilize one protein do not necessarily exert equivalent effects on others, and comparative studies have repeatedly reported inconsistent or even conflicting structural correlates of thermostability. Thus, although general structural characteristics explain part of proteome-wide variation in thermal stability, it remains unclear whether paralogs with extreme sequence-adjusted thermal differences share consistent patterns of structural remodelling. It also remains untested whether duplication mode independently predicts extreme thermal divergence after controlling for sequence similarity, or whether such divergence correlates more closely with functional specialization and differences in cellular context.

Here, we reanalyzed a high-confidence thermal-stability atlas comprising 3917 *Arabidopsis* proteins and benchmarked the resulting T_m_ measurements against another independent *Arabidopsis* meltome dataset [10,20]. We integrated gene-family annotations, paralog relationships, duplication modes, pairwise sequence similarity, structure-derived features, Gene Ontology annotations and subcellular-context information to dissect thermal stability across complementary organizational scales. We first quantified between-family and within-family thermal variation and tested whether proteins from the same family display higher thermal similarity than size-matched random protein groups. We then modelled the relationship between sequence similarity and pairwise ΔT_m_, defining a sequence-adjusted thermal residual to identify paralog pairs with exceptionally high or low thermal divergence relative to sequence-derived expectations. Mixed-effects models separated contributions from gene-family identity and measurable protein-structural properties, while pairwise models assessed the independent effects of duplication mode, structural divergence, functional-annotation distance and cellular-context divergence.

## 2. Results

### 2.1. A High-Confidence Thermal Atlas Reveals Gene-Family-Associated Patterns of Protein Thermal Stability

**Figure 1:**
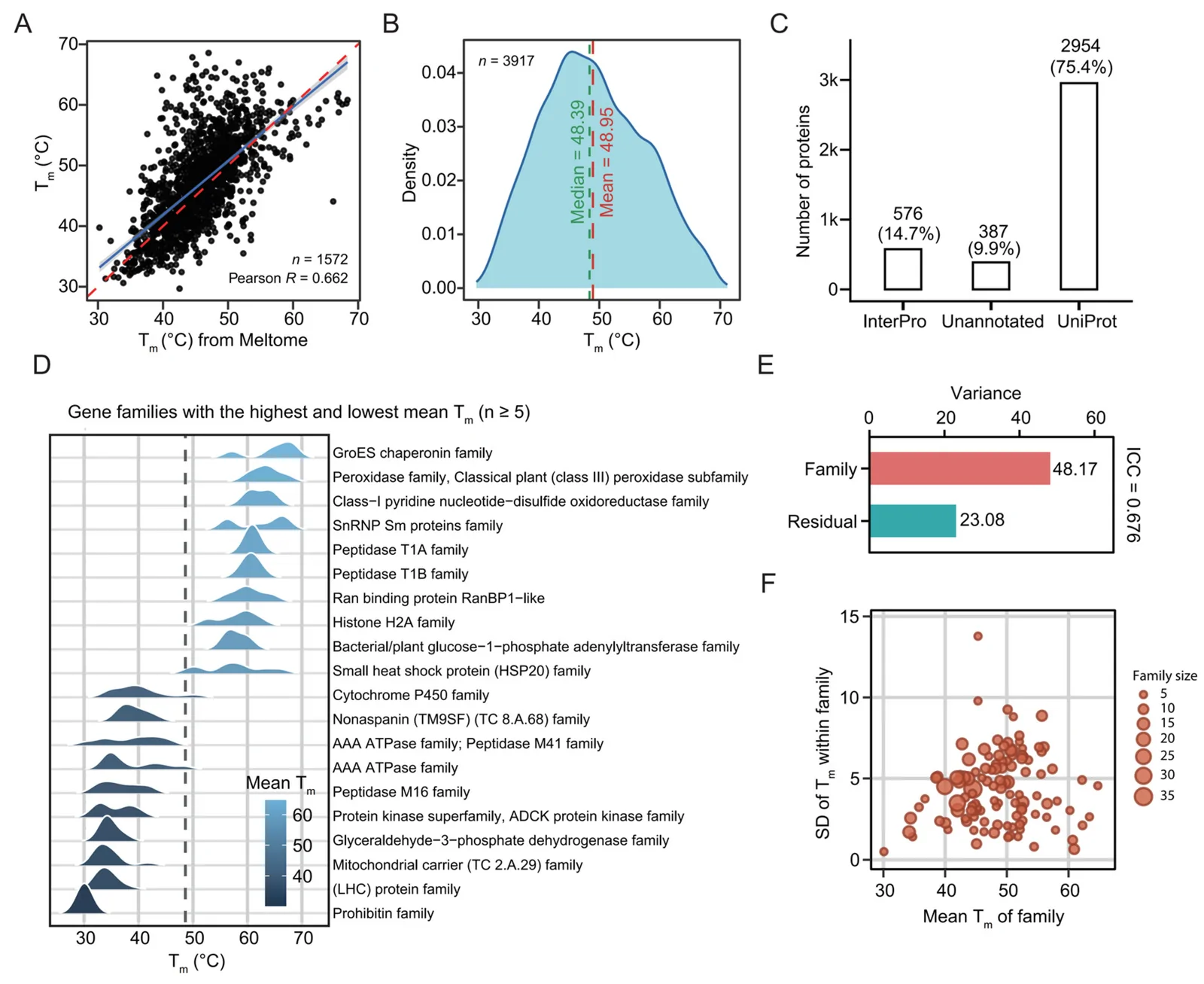
A high-confidence *Arabidopsis* thermal atlas reveals gene-family-associated patterns of protein stability. (**A**) Comparison of protein melting temperatures (T_m_) between two previously published *Arabidopsis* thermal-proteome datasets for 1572 matched proteins. The blue diagonal denotes the identity line (*y* = *x*), and the red line represents the linear regression fit. Pearson’s correlation coefficient is shown. (**B**) Density distribution of T_m_ values for the 3917 proteins included in the *Arabidopsis* thermal atlas. The green and red dashed lines indicate the median and mean T_m_, respectively. (**C**) Sources of gene-family annotation for proteins in the thermal atlas. Numbers and percentages are shown above the bars. (**D**) Ridgeline distributions of T_m_ for gene families with the highest and lowest mean T_m_. Only families containing at least five proteins with valid T_m_ measurements were included. Ridge color represents the mean T_m_ of each family, and the vertical dashed line indicates the median T_m_ of the complete thermal atlas. (**E**) Variance components estimated using a random-intercept mixed-effects model with gene family included as the grouping factor. Bars show the between-family variance (48.17) and within-family residual variance (23.08). The corresponding intraclass correlation coefficient (ICC) was 0.676. (**F**) Relationship between the mean T_m_ and within-family standard deviation of T_m_ across gene families containing at least five measured proteins. Each point represents one family, and point size is proportional to the number of detected family members.

To assess the consistency of previously reported *Arabidopsis* protein melting temperatures, we compared two independent thermal proteome datasets [10,20]. Among the 1572 proteins quantified in both datasets, the two measurements showed a clear positive correlation (Pearson’s *R* = 0.662), supporting the overall reproducibility of protein thermal stability estimates across experimental platforms (Figure 1A). After data quality control, melting temperatures (Tm) were determined for 3917 proteins. The resulting T_m_ values followed a broad, approximately unimodal distribution, with a mean of 48.95 °C and a median of 48.39 °C, revealing substantial proteome-wide variation in thermal stability (Figure 1B).

To facilitate gene-family-level analyses, detected proteins were annotated using UniProt (https://www.uniprot.org/) and InterPro 109.0 (https://www.ebi.ac.uk/interpro/). UniProt provided family assignments for 2954 proteins (75.4%), while InterPro delivered supplementary annotations for 576 proteins (14.7%) lacking UniProt family classification. In total, 3530 proteins (90.1% of the quantified thermal proteome) were assigned to a protein family, while 387 proteins (9.9%) remained unannotated (Figure 1C). Analysis of families containing at least five proteins with valid T_m_ measurements uncovered striking divergence in thermal stability distributions (Figure 1D and Table S1). Families with relatively high mean T_m_ included the GroES chaperonin, classical plant class III peroxidase, SnRNP Sm protein, histone H2A and small heat shock protein families. In contrast, multiple membrane-associated or conformationally dynamic protein groups, including cytochrome P450, mitochondrial carrier and light-harvesting complex protein families, displayed comparatively low mean T_m_ values. These differences constitute family-associated thermal patterns and may additionally arise from systematic variation in protein localization, cofactor binding, complex assembly, and dependence on the native membrane environment.

We further quantified the degree to which protein thermal stability is shaped at the gene-family level using a random-intercept linear mixed-effects model. The estimated between-family variance was 48.17, and the residual within-family variance was 23.08, yielding an intraclass correlation coefficient (ICC) of 0.676 (Figure 1E). Accordingly, the ICC indicated that approximately 67.6% of the total observed variation in T_m_ could be attributed to differences across annotated protein families, demonstrating strong family-level structuring within the thermal proteome. Nevertheless, substantial heterogeneity persisted within individual families. Comparisons of family-average T_m_ values and within-family standard deviations revealed that families with comparable mean thermal stability could differ considerably in the magnitude of member-to-member variation (Figure 1F). Certain families harboured tightly clustered T_m_ values, consistent with robust conservation of thermal behavior, whereas others displayed broad distributions pointing to marked intra-family thermal heterogeneity. Together, these results suggest that gene-family membership may serve as a major factor shaping protein thermal stability, while considerable thermal diversity still exists among paralogs within many gene families.

### 2.2. Conservation and Divergence of Thermal Stability Among Arabidopsis Paralogs

Gene-family membership shapes protein thermal stability in *Arabidopsis,* yet thermal heterogeneity varies widely among different gene families. It remains unclear whether intra-family thermal heterogeneity originates from random proteome fluctuation or non-random evolutionary divergence between paralogous proteins. To address this question, we characterized thermal stability conservation and divergence among paralogs by comparing observed within-family protein differences against matched random controls and exploring associations with sequence similarity and gene duplication mode.

**Figure 2:**
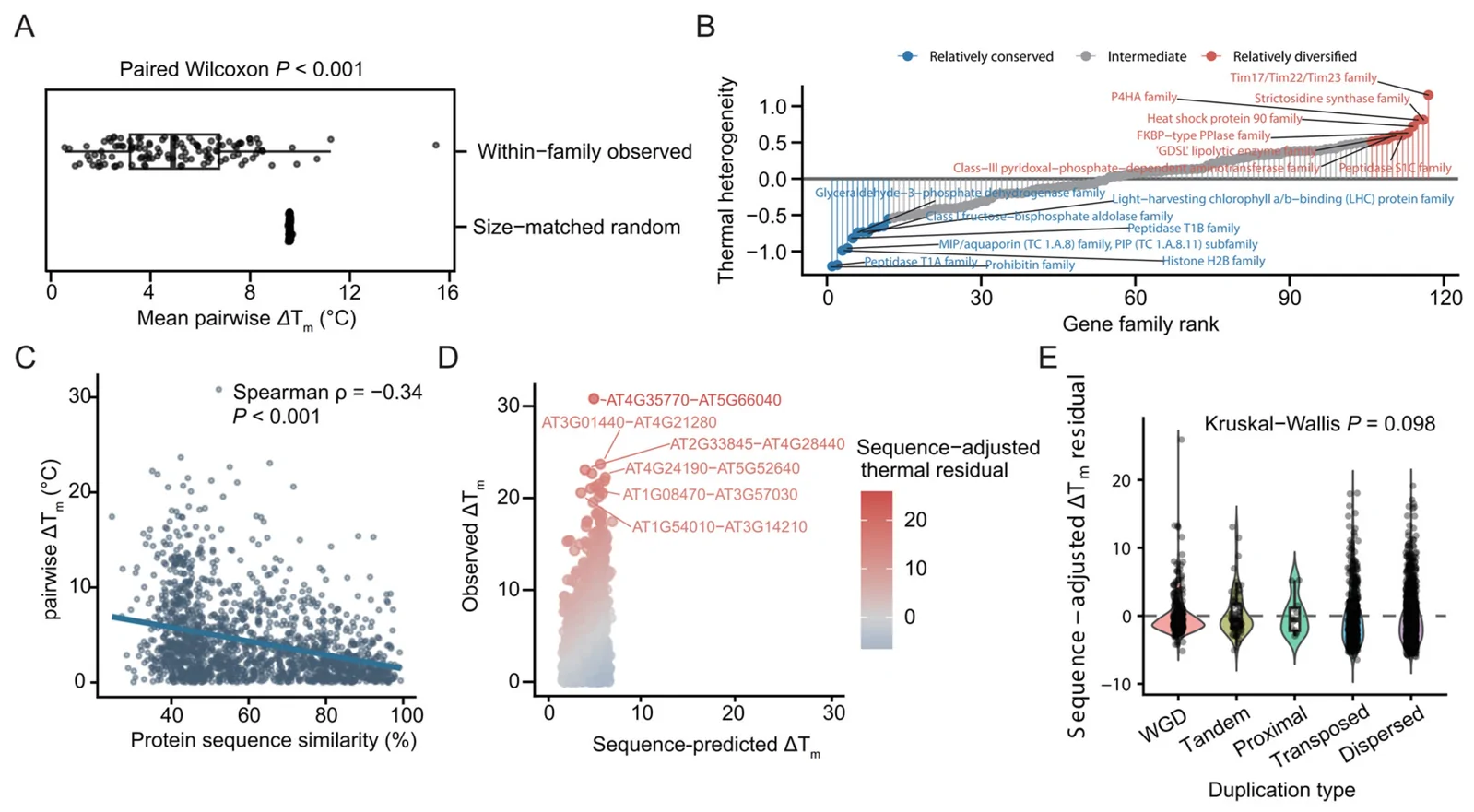
*Arabidopsis* paralogs exhibit family-dependent patterns of thermal conservation and divergence. (**A**) Comparison of the observed mean pairwise absolute difference in melting temperature (ΔT_m_) within gene families and the corresponding expectation from size-matched random protein groups. For each family containing at least five proteins with valid T_m_ measurements, the observed mean pairwise ΔT_m_ was calculated and compared with the mean value obtained from repeated random sampling of an equal number of proteins from the background thermal proteome. Each point represents one gene family; boxplots show the median and interquartile range. Statistical significance was assessed using a two-sided paired Wilcoxon signed-rank test. (**B**) Relative thermal heterogeneity across gene families. Families were ranked according to family-size-adjusted within-family thermal heterogeneity, calculated as the residual from the relationship between detected family size and mean pairwise ΔT_m_. Families in the lower and upper 10% of the residual distribution were classified as relatively conserved and relatively diversified, respectively, whereas the remaining families were classified as intermediate. Selected families at the extremes of the distribution are labelled. (**C**) Relationship between protein-sequence similarity and pairwise thermal divergence among paralogous proteins. Each point represents one paralog pair, and pairwise ΔT_m_ was calculated as the absolute difference between the two measured T_m_ values. The blue curve represents the fitted relationship between sequence similarity and ΔT_m_. Spearman’s rank correlation coefficient and its associated *p* value are shown. (**D**) Identification of paralog pairs with thermal divergence that deviates from the expectation based on sequence similarity. The *x*-axis shows sequence-predicted ΔT_m_, and the *y*-axis shows the observed ΔT_m_. Point colour indicates the sequence-adjusted thermal residual, calculated as observed ΔT_m_ minus sequence-predicted ΔT_m_. (**E**) Distribution of sequence-adjusted thermal residuals among paralog pairs assigned to whole-genome duplication (WGD), tandem, proximal, transposed and dispersed duplication classes. Violin plots show the residual distributions, embedded boxplots indicate the median and interquartile range, and individual points represent paralog pairs. The dashed horizontal line indicates a residual of zero. Differences among duplication classes were assessed using a Kruskal–Wallis test.

For each gene family containing at least five proteins with valid melting-temperature measurements, we calculated the mean pairwise absolute difference in T_m_ and compared it with the expectations derived from size-matched random protein groups. The observed within-family pairwise ΔT_m_ values were substantially lower than the corresponding randomized expectations (paired Wilcoxon test, *p* < 0.001; Figure 2A), demonstrating that proteins belonging to the same family generally exhibit greater similarity in thermal stability than unrelated proteins. Nevertheless, the degree of such conservation varied markedly across families. After accounting for the relationship between family member count and within-family thermal variation, families could be classified into relatively conserved, intermediate, and relatively diversified groups (Figure 2B and Table S2). Families with low thermal heterogeneity included histone H2B, peptidase T1A/T1B and light-harvesting chlorophyll (a/b)-binding protein families. In contrast, the Tim17/Tim22/Tim23, P4HA, HSP90 and FKBP-type peptidyl-prolyl isomerase families displayed broad thermal variation. These categories represent relative positions within the observed distribution of families and do not constitute formal evidence that the diversified families are significantly more heterogeneous than random protein assemblies.

We next investigated whether thermal divergence between paralogous correlates with primary-sequence conservation. Pairwise ΔT_m_ showed a negative correlation with protein sequence similarity (Spearman’s ρ = −0.34, *p* < 0.001; Figure 2C), indicating that closely related paralogs typically retain more similar thermal stability. However, substantial scattering of ΔT_m_ was observed for protein pairs with comparable sequence similarity, revealing that sequence conservation only partially explains the observed thermal variation. We therefore modelled the expected relationship between sequence similarity and pairwise ΔT_m_ and defined a sequence-similarity-adjusted thermal residual as the deviation between observed and predicted ΔT_m_. Most paralog pairs clustered near the model’s predicted values, while a subset exhibited strongly positive residuals, corresponding to far greater thermal divergence than would be anticipated from sequence similarity alone (Figure 2D). Conversely, negative residuals identified pairs whose thermal stability remained more similar than predicted despite sequence divergence. Finally, the distributions of sequence-adjusted thermal residuals did not differ significantly among paralog pairs originating from WGD, tandem, proximal, transposed, and dispersed duplication (Kruskal–Wallis, *p* = 0.098; Figure 2E and Table S3). Together, these results show that thermal stability is broadly conserved among *Arabidopsis* paralogs, yet a subset of protein pairs displays pronounced thermal divergence. This divergence cannot be fully explained by primary-sequence similarity and exhibits no statistically significant association with duplication mode.

### 2.3. Gene-Family Identity Dominates Protein Thermal Stability, with Additional Contributions from Protein Architecture

**Figure 3:**
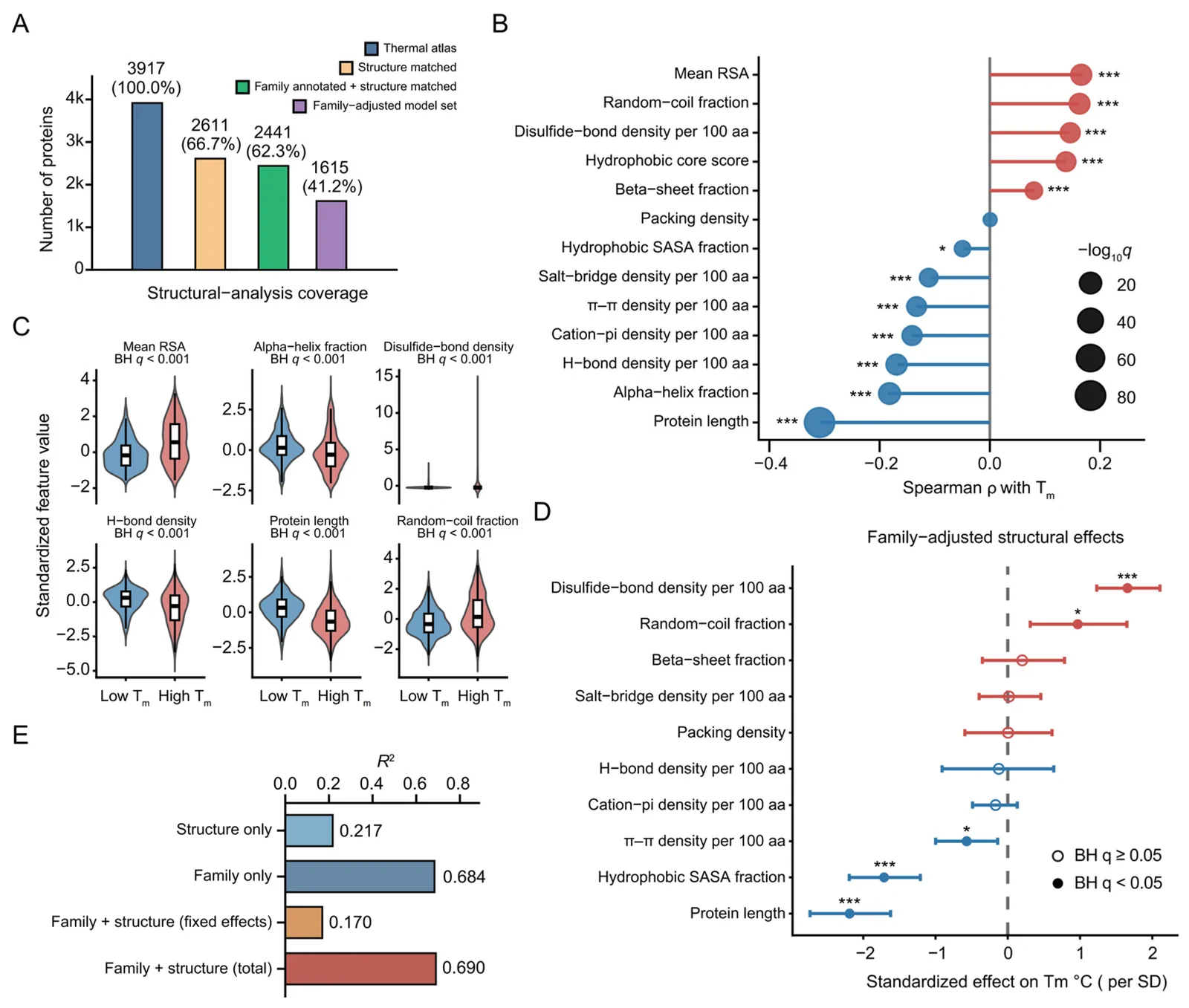
Gene-family identity dominates protein thermal stability, with additional contributions from protein architecture. (**A**) Coverage of proteins retained at successive stages of the structural analysis. (**B**) Proteome-wide associations between individual structural features and melting temperature (T_m_). Points represent Spearman correlation coefficients, horizontal segments extend from zero to the corresponding coefficient, and point size is proportional to −log_10_(*q*), where *q* denotes the Benjamini–Hochberg-adjusted *p* value. Red and blue indicate positive and negative correlations, respectively. Asterisks indicate BH-adjusted significance: * *q* < 0.05 and *** *q* < 0.001. RSA, relative solvent accessibility; SASA, solvent-accessible surface area; aa, amino acids. (**C**) Comparison of selected structural features between proteins in the lower and upper T_m_ quintiles. Violin plots show the feature distributions, and embedded boxplots indicate the median and interquartile range. Feature values were standardized across proteins before visualization. Statistical significance was evaluated using two-sided Wilcoxon rank-sum tests followed by Benjamini–Hochberg correction. (**D**) Independent associations between structural features and T_m_ estimated using a multivariable linear mixed-effects model with gene family included as a random intercept. Continuous predictors were standardized before model fitting. Points show coefficient estimates and horizontal bars indicate 95% confidence intervals. Filled and open points denote BH-adjusted *q* < 0.05 and *q* > 0.05, respectively. Red and blue indicate positive and negative coefficient estimates. Asterisks indicate BH-adjusted significance: * *q* < 0.05, and *** *q* < 0.001. (**E**) Variance explained by structural features and gene-family identity. The structure-only model is summarized using ordinary *R*^2^; the family-only model is represented by conditional *R*^2^; and the family-plus-structure model is shown using marginal *R*^2^, representing fixed structural effects, and conditional *R*^2^, representing the combined fixed and family-level effects.

To determine whether protein structural properties predict thermal stability independently of gene-family membership, we integrated the *Arabidopsis* thermal proteome database with protein structure features. Among the 3917 proteins with valid T_m_ measurements, 2611 proteins could be matched to structural information, and 2441 additionally possessed gene-family annotations. We retained 1615 proteins originating from multi-member gene families for the family-adjusted multivariable modelling (Figure 3A and Table S4). Univariate analyses detected significant associations between T_m_ and multiple structural descriptors (Figure 3B). Mean relative solvent accessibility, random-coil fraction, disulfide-bond density, hydrophobic-core score and β-sheet fraction were positively associated with T_m_. By contrast, protein length, α-helix fraction, hydrophobic solvent-accessible surface area, and several residue-interaction densities exhibited negative correlations. Consistent with these trends, proteins within the high T_m_ group differed significantly from low T_m_ proteins in mean RSA, protein length, α-helix fraction, random-coil fraction, hydrogen-bond density and disulfide-bond densities (BH-adjusted *q* < 0.001; Figure 3C). These proteome-wide comparisons demonstrate that proteins with distinct thermal stability occupy separate regions within structural-feature space, although these raw differences may partially arise from uneven family composition.

We therefore constructed multivariable mixed-effects models, incorporating gene family as a random intercept and standardized structural characteristics as fixed effects. After adjusting for family membership and structural variables, disulfide-bond density and random-coil fraction remained independently and positively associated with T_m_. Protein length, hydrophobic SASA fraction and π-π interaction density showed significant negative associations (BH-adjusted *q* < 0.05; Figure 3D). The positive effect of disulfide-bond density and the negative effects of protein length and exposed hydrophobic surface broadly align with established models: covalent cross-linking, molecular size, and hydrophobic burial jointly shape protein thermal stability. In contrast, associations for random-coil fraction and π-π density appear context-dependent and should not be generalized as universal stabilizing or destabilizing mechanisms [20,52,53,54].

After multivariable adjustment, packing density, β-sheet fraction, salt-bridge density, hydrogen-bond density and cation-π density exhibited no statistically significant independent effects on T_m_. Structural features alone explained 21.7% of total T_m_ variation, whereas the family-only mixed model yielded a conditional *R*^2^ of 0.684 (Figure 3E). Incorporating structural characteristics significantly improved model performance relative to the family-only model (likelihood-ratio test, *p* < 0.001), yet the conditional *R*^2^ increased only modestly from 0.684 to 0.690. Collectively, these results establish that gene-family identity constitutes the primary source of thermal-stability variation across the *Arabidopsis* proteome, while protein architectural traits provide statistically detectable yet comparatively limited supplementary explanatory power.

### 2.4. Exceptional Thermal Divergence Among Arabidopsis Paralogs Lacks a Uniform Structural Signature

**Figure 4:**
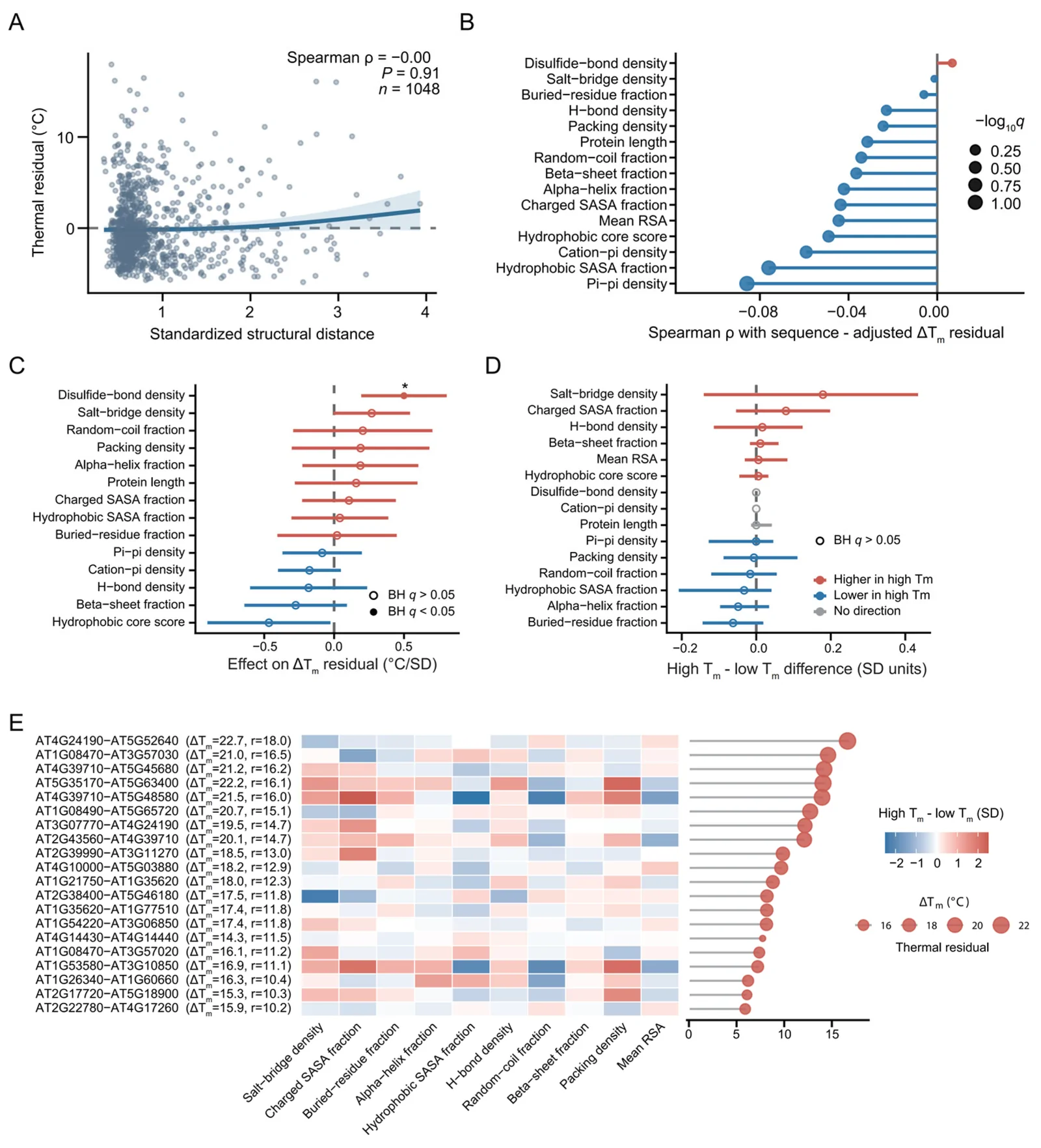
Exceptional thermal divergence among *Arabidopsis* paralogs lacks a uniform structural signature. (**A**) Relationship between overall structural divergence and sequence-adjusted thermal divergence among 1048 paralog pairs with complete structural information. Composite structural distance was calculated from standardized pairwise differences in protein length, secondary structure composition, relative solvent accessibility, solvent-accessible surface area, residue burial, packing properties, and intramolecular interaction densities. The solid curve shows the fitted trend and the shaded region indicates the 95% confidence interval. The horizontal dashed line denotes a thermal residual of zero. Spearman’s correlation coefficient, *p* value, and sample size are shown. (**B**) Associations between individual absolute structural differences and sequence-adjusted thermal residuals. Points represent Spearman correlation coefficients, horizontal segments extend from zero to the corresponding coefficient, and point size is proportional to −log_10_(*q*), where *q* denotes the Benjamini–Hochberg-adjusted *p* value. Red and blue indicate positive and negative correlations, respectively. RSA, relative solvent accessibility; SASA, solvent-accessible surface area; aa, amino acids. (**C**) Independent associations between structural differences and sequence-adjusted thermal divergence estimated using a multivariable linear model that additionally included duplication type. Continuous structural predictors were standardized before model fitting, and standard errors were two-way clustered by the two genes forming each paralog pair to account for repeated gene participation. Points show coefficient estimates and horizontal bars indicate 95% confidence intervals. Filled and open points denote BH-adjusted *q* < 0.05 and *q* > 0.05, respectively. Red and blue indicate positive and negative coefficient estimates. Asterisks indicate * *q* < 0.05. (**D**) Directional structural differences among paralog pairs in the upper 10% of the sequence-adjusted thermal-residual distribution. Points indicate median standardized differences, and horizontal bars show bootstrap 95% confidence intervals. Statistical significance was assessed using two-sided one-sample Wilcoxon signed-rank tests followed by Benjamini–Hochberg correction. Positive and negative values indicate features with higher and lower values, respectively, in the paralog with higher T_m_. (**E**) Structural-remodeling profiles of the paralog pairs with the largest positive thermal residuals. Each row represents one paralog pair, with the observed pairwise ΔT_m_ and sequence-adjusted thermal residual indicated in the row label. Heatmap colors represent standardized higher T_m_ minus lower T_m_ differences, with red and blue indicating higher and lower feature values, respectively. The accompanying dot plot shows the sequence-adjusted thermal residual for each pair, and point size represents the observed pairwise ΔT_m_.

To determine whether thermal divergence between paralogous proteins could be explained by differences in protein architecture, we integrated sequence-adjusted thermal residuals with pairwise structural comparisons. We computed a composite structural distance from standardized differences in protein length, secondary-structure composition, solvent accessibility, residue burial, packing properties and intramolecular interaction densities. However, overall structural distance showed no association with sequence-adjusted thermal divergence across the 1048 paralog pairs with complete structural information (Spearman’s ρ ≈ 0.00, *p* = 0.91; Figure 4A and Table S5). Consistent with this, correlations between individual absolute structural differences and thermal residuals were generally weak, with all effect sizes remaining small and none reaching significance after Benjamini–Hochberg correction (Figure 4B). These results indicate that global structural dissimilarity alone does not systematically account for thermal differences that exceed expectations based on sequence similarity.

We next fitted a multivariable model incorporating standardized structural differences and duplication type, with standard errors clustered by both genes to account for repeated participation of individual genes in multiple paralog pairs. Among the structural variables examined, only disulfide-bond density difference exhibited a significant positive association with sequence-adjusted thermal divergence after multiple-testing correction (Figure 4C). Thus, paralog pairs with greater differences in disulfide-bond density tended to display larger T_m_ differences than predicted from their sequence similarity. By contrast, differences in protein length, packing density, hydrophobic-core score, solvent exposure, secondary-structure composition and other residue-interaction densities showed no significant independent associations. We further examined paralog pairs in the top decile of the thermal-residual distribution to assess whether the higher T_m_ members shared a common direction of structural remodeling. Although modest trends were observed for several features, none of the high T_m_ versus low T_m_ contrasts remained significant after multiple-testing correction (Figure 4D). Moreover, the most strongly divergent paralog pairs displayed heterogeneous combinations of structural changes, with no consistent feature signature shared across candidates (Figure 4E). Together, these findings suggest that exceptional thermal divergence among *Arabidopsis* paralogs is not governed by a universal structural remodeling program, but instead arises through heterogeneous, pair-specific changes, with altered disulfide-bond density representing the clearest independent structural correlate.

### 2.5. Functional Context Stratifies Exceptional Thermal Divergence and Prioritizes Candidate Paralog Pairs

**Figure 5:**
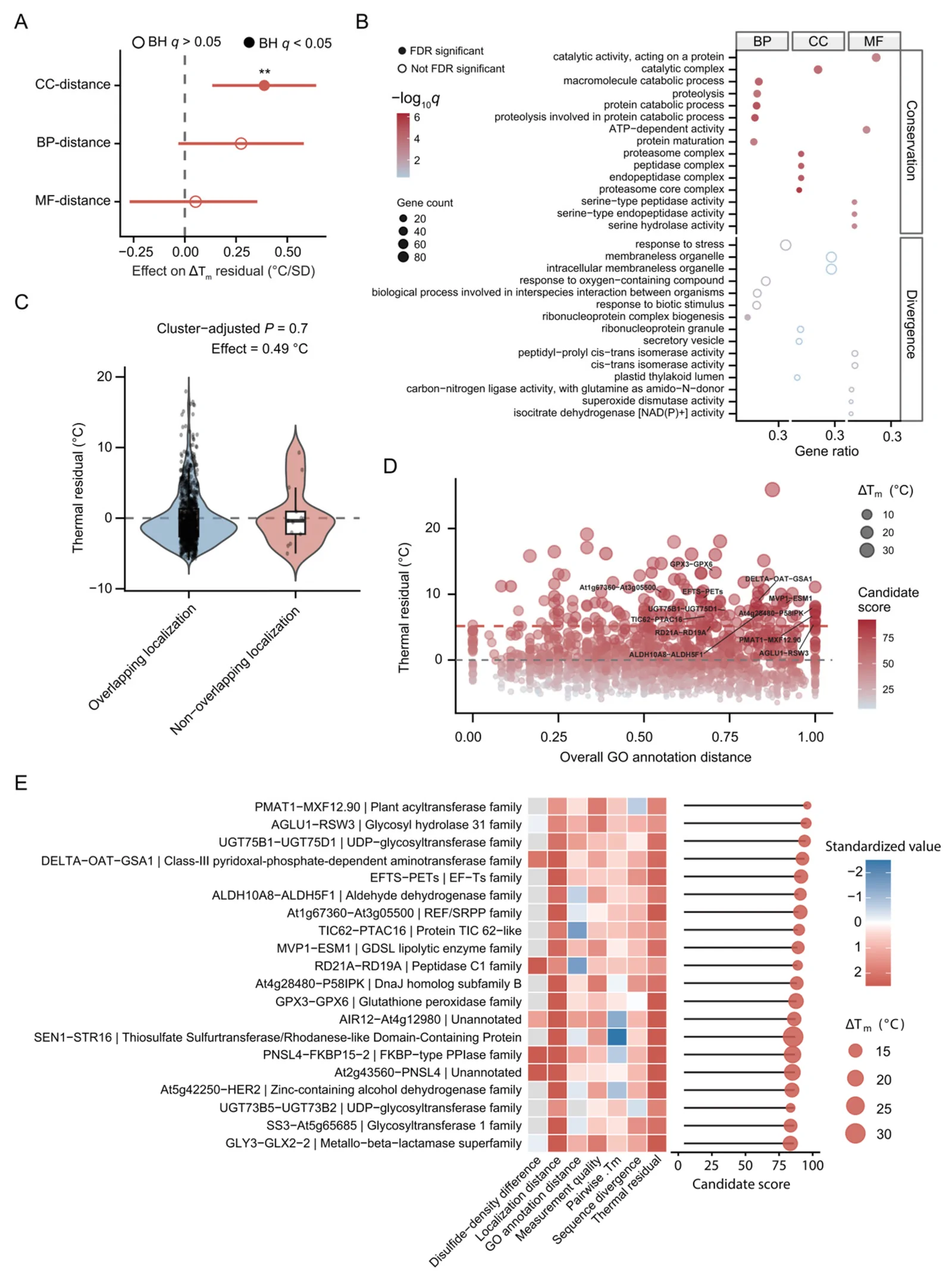
Functional context stratifies exceptional thermal divergence and prioritizes candidate *Arabidopsis* paralog pairs. (**A**) Associations between functional-annotation divergence and sequence-adjusted thermal residuals. Pairwise distances in Gene Ontology biological process (BP), molecular function (MF), and cellular component (CC) were calculated as one minus the Jaccard similarity between the corresponding GO-term sets. Points show estimated effects on the thermal residual per standard deviation increase in GO distance, and horizontal bars indicate 95% confidence intervals. Filled and open points denote Benjamini–Hochberg-adjusted *q* < 0.05 and *q* > 0.05, respectively. Asterisks indicate ** *q* < 0.01. BP, biological process; CC, cellular component; MF, molecular function. (**B**) GO enrichment analysis of proteins from paralog pairs exhibiting exceptional thermal conservation or exceptional thermal divergence, defined as the lower and upper 10% of the sequence-adjusted thermal-residual distribution, respectively. The top enriched terms in each ontology and residual class are shown. Point size represents the number of genes assigned to each term, colour indicates −log_10_(*q*), and filled and open points denote FDR-significant and non-significant terms, respectively. (**C**) Comparison of sequence-adjusted thermal residuals between paralog pairs with overlapping and non-overlapping broad subcellular-localization annotations. Violin plots show the distributions, embedded boxplots indicate the median and interquartile range, and points represent individual paralog pairs. (**D**) Integrated prioritization landscape of *Arabidopsis* paralog pairs. The *x*-axis shows the overall GO annotation distance, calculated as the mean of available BP, MF, and CC distances, and the *y*-axis shows the sequence-adjusted thermal residual. Point size represents the observed pairwise ΔT_m_, whereas colour indicates the composite candidate score. The candidate score integrates percentile-ranked thermal residual, observed ΔT_m_, overall GO distance, disulfide-bond-density difference, and measurement quality, with weights of 0.40, 0.25, 0.15, 0.10, and 0.10, respectively. The red dashed line marks the upper 10% threshold of the thermal-residual distribution, and the grey dotted line indicates zero residual. Selected high-ranking candidate pairs are labelled. (**E**) Integrated feature profiles of the top-ranked paralog pairs. Each row represents one candidate pair, labelled by gene names and protein-family annotation. Heatmap colours show standardized values for disulfide-bond-density difference, localization distance, overall GO annotation distance, measurement quality, observed pairwise ΔT_m_, sequence divergence, and thermal residual; red and blue indicate values above and below the dataset mean, respectively. The accompanying lollipop plot shows the composite candidate score, and point size represents the observed pairwise ΔT_m_.

Because exceptional thermal divergence was not explained by a uniform structural signature, we next examined whether divergence in functional annotation and cellular context associated with sequence-adjusted thermal residuals. In a multivariable model accounting for duplication type and repeated gene participation across paralog pairs, GO cellular-component distance showed a significant positive association with thermal residuals (BH-adjusted *q* < 0.01; Figure 5A and Table S6), whereas biological-process and molecular-function distances were not significant. Thus, paralog pairs assigned to more distinct cellular components tended to display larger T_m_ differences than expected from their sequence similarity. Functional enrichment analysis further revealed contrasting biological contexts at the two extremes of the residual distribution (Figure 5B and Table S7). Proteins from exceptionally thermally conserved pairs were significantly enriched in protein catabolic processes, proteasome and peptidase complexes, endopeptidase activity, and serine-type peptidase or hydrolase activity, suggesting that components of the protein-quality-control machinery are subject to comparatively strong constraints on thermal stability. In contrast, proteins from exceptionally divergent pairs were associated with a broader set of terms, including responses to stress and oxygen-containing compounds, membraneless organelles, ribonucleoprotein-complex biogenesis, secretory vesicles, and the plastid thylakoid lumen, although most of these enrichments did not remain significant after multiple-testing correction. Consistent with this heterogeneity, a binary comparison based on broad subcellular-localization overlap showed no significant difference in thermal residuals between paralog pairs with overlapping versus non-overlapping localization assignments (cluster-adjusted *p* = 0.7, estimated effect = 0.49 °C; Figure 5C). These results indicate that fine-grained cellular-component divergence, rather than a simple switch in broad organellar localization, is more closely associated with exceptional thermal divergence.

We therefore integrated thermal residuals, observed pairwise ΔT_m_, GO annotation distance, localization distance, disulfide-bond-density difference, and measurement quality to prioritize candidate paralog pairs for mechanistic validation (Figure 5D,E). Several high-ranking pairs combined large positive residuals with substantial functional divergence. For example, GPX3-GPX6, belonging to the glutathione peroxidase family, showed one of the strongest combinations of thermal divergence and candidate score, making this pair particularly suitable for testing whether redox-related functional specialization is accompanied by altered protein stability. The glycosyltransferase pairs UGT75B1-UGT75D1 and UGT73B5-UGT73B2 were also highly ranked, suggesting that diversification within UDP-glycosyltransferase families may be accompanied by pronounced thermal differentiation. Similarly, DELTA-OAT-GSA1, annotated as a class III pyridoxal-phosphate-dependent aminotransferase pair, and ALDH10A8-ALDH5F1, belonging to the aldehyde dehydrogenase family, combined high thermal residuals with marked functional or cellular-context differences. Additional candidates included MVP1-ESM1, TIC62-PTAC16, RD21A-RD19A, PNSL4-FKBP15-2, and PMAT1-MXF12.90, which represent distinct functional families and exhibited different combinations of sequence divergence, localization distance, structural change, and thermal residual. The heterogeneous profiles of these candidates reinforce the conclusion that exceptional thermal divergence does not arise through a single common mechanism but instead reflects paralog-specific combinations of structural, functional, and cellular-context changes. Collectively, these analyses identify a focused set of biologically interpretable paralog pairs for subsequent validation by recombinant-protein thermal profiling, activity assays, or targeted mutagenesis.

## 3. Discussion

By integrating lysate-based thermal profiling with gene-family relationships, sequence similarity, duplication history, structural descriptors, and functional annotations, this study provides a hierarchical framework for understanding protein thermal stability in *Arabidopsis thaliana*. The thermal atlas encompasses 3917 proteins and shows moderate concordance with an independent *Arabidopsis* dataset, supporting the general reproducibility of the measurements. Because the experiments were performed in lysates rather than intact cells, the measured T_m_ values primarily reflect the biochemical properties of proteins under a standardized extract environment, including intrinsic folding stability and any interactions, ligands, or complexes retained after lysis. These values should therefore not be interpreted as direct measurements of protein stability within living cellular compartments.

Gene-family identity emerged as the predominant determinant of thermal stability. The intraclass correlation coefficient of 0.676 indicates that a substantial proportion of T_m_ variation occurs between protein families rather than among members of the same family. This observation is consistent with the conservation of sequence, domain architecture, tertiary fold, catalytic organization, and oligomeric state within protein families. Similar thermal behavior among evolutionarily related proteins and members of the same molecular complex has also been reported in previous thermal-proteome studies [10,20,23,27]. Nevertheless, family identity represents a combination of correlated molecular properties rather than a single causal factor. The strong family effect therefore indicates that evolutionary history constrains thermal stability, while the specific sequence or structural determinants underlying this constraint may differ among families.

Thermal stability was generally more similar within families than expected from random protein groups, yet substantial variation remained among individual paralogs. Pairwise ΔT_m_ decreased with increasing sequence similarity, indicating that sequence divergence contributes to thermal differentiation. However, the moderate strength of this relationship suggests that sequence similarity alone is insufficient to predict thermal behavior. Changes at structurally or functionally critical residues may have disproportionately large effects, whereas compensatory substitutions may preserve stability despite substantial sequence divergence. By modeling the expected relationship between sequence similarity and ΔT_m_, we identified paralog pairs that were more thermally divergent or conserved than expected. These residuals provide a useful relative measure, although they depend on the fitted global relationship and may not fully capture family-specific evolutionary trajectories or domain rearrangements.

After sequence adjustment, duplication mode was not significantly associated with thermal divergence. This suggests that broad categories such as whole-genome, tandem, proximal, transposed, and dispersed duplication are not strong general predictors of paralog thermal differentiation in the current dataset. This result should not be interpreted as excluding an effect of duplication history. Duplication classes differ in evolutionary age, family composition, and selective constraints, and some categories contained relatively few measurable protein pairs. More extensive datasets may reveal duplication effects within particular families or evolutionary periods.

Structural properties explained a detectable but comparatively limited proportion of proteome-wide T_m_ variation. Protein length, solvent exposure, secondary-structure composition, hydrophobic surface properties, and disulfide-bond density were among the features associated with thermal stability, broadly consistent with established contributions of packing, compactness, and intramolecular interactions to protein thermostability [49,50,51,52]. However, adding these structural descriptors to a family-based model increased the conditional *R*^2^ only modestly, from 0.684 to 0.690. This suggests that much of the structural signal is already captured by gene-family identity. It also indicates that global structural descriptors alone cannot fully explain protein thermal behavior. Local changes near ligand-binding sites, catalytic residues, flexible loops, or oligomerization interfaces may be more important than differences in whole-protein averages.

Consistent with this interpretation, exceptional thermal divergence among paralogs was not accompanied by a uniform pattern of structural remodeling. Composite structural distance showed little relationship with sequence-adjusted thermal residuals, and the most divergent pairs exhibited heterogeneous combinations of structural changes. Differences in disulfide-bond density provided the clearest independent association, although the estimated effect was modest. Disulfide bonds can restrict conformational flexibility and enhance stability, making this relationship biologically plausible, but predicted disulfide density may not accurately represent disulfide formation under the lysate conditions used here. This feature should therefore be considered a candidate mechanism for experimental testing rather than a general explanation of paralog thermal divergence.

Functional annotations provided an additional perspective. Divergence in GO cellular-component annotation was positively associated with sequence-adjusted thermal residuals, whereas biological-process and molecular-function distances showed weaker effects. In a lysate-based experiment, this association does not demonstrate that different intracellular environments directly caused the observed thermal differences. Instead, cellular-component divergence may act as a proxy for broader functional specialization, including differences in complex membership, membrane association, domain organization, interaction partners, or biochemical roles that remain partly reflected after protein extraction. The lack of a significant difference between paralog pairs with overlapping and non-overlapping broad localization categories further suggests that coarse organelle-level assignments are insufficient to explain thermal divergence.

Exceptionally conserved paralogs were enriched in proteolysis, peptidase complexes, and protein-catabolic functions. Proteins involved in protein-quality control often depend on conserved catalytic architecture and coordinated complex assembly, which may constrain their thermal properties. By contrast, exceptionally divergent paralogs were distributed across a broader and less statistically robust range of functions, including stress responses and plastid- or vesicle-associated processes. This pattern is consistent with multiple family-specific routes to thermal divergence rather than a single enriched biological pathway.

Our classification of gene families into conserved, intermediate and diversified groups raises a key question about the mechanisms underlying this heterogeneity in intra-family thermal conservation. Disulfide-bond density and cellular-component divergence explain only part of paralog thermal divergence and cannot fully account for family-level differences in thermal homogeneity. Functional constraints, expression patterns, structural complexity and evolutionary age may all be involved, and disentangling their contributions will require integrated transcriptomic and evolutionary rate data in future work.

The integrated analysis prioritized several paralog pairs for further investigation. GPX3-GPX6 may be useful for examining whether redox-related functional specialization is accompanied by altered intrinsic stability. As glutathione peroxidases, they participate in cellular reactive oxygen species scavenging under abiotic stress [55,56]; their thermal divergence might influence ROS-homeostasis maintenance during high-temperature exposure. The UDP-glycosyltransferase pairs, UGT75B1-UGT75D1 and UGT73B5-UGT73B2, are strong candidates for connecting thermal divergence to stress-related functional specialization. Plant UDP-glycosyltransferases regulate hormone homeostasis, secondary-metabolite turnover and xenobiotic detoxification through glycosylation. UGT75B1 mediates abscisic acid glycosylation under abiotic stress [57], while UGT73B5 contributes to cellular redox homeostasis during biotic-stress responses [58]. The notable thermal divergence between these paralogs may partly arise from differences in substrate specificity and ligand-binding properties that modify protein conformational stability. These glycosyltransferases could be targets for thermotolerance engineering, since tuning their thermal stability may preserve cellular detoxification and hormone regulation under high-temperature conditions. The Δ-OAT-GSA1 pair belongs to class III pyridoxal-phosphate-dependent aminotransferases and exemplifies thermal divergence among metabolically distinct enzymes. DELTA-OAT drives ornithine-dependent proline biosynthesis for stress-related osmoprotection [59], whereas GSA1 contributes to tetrapyrrole and chlorophyll biosynthesis as part of core photosynthetic metabolism [60]. Their differing thermal stabilities may reflect distinct requirements for enzyme activity under normal versus heat-stressed conditions. Modifying the thermal stability of such metabolic enzymes may help sustain photosynthetic performance and boost protective metabolite accumulation during heat stress. ALDH10A8-ALDH5F1 extends these comparisons to additional cofactor-dependent detoxification enzymes. Members of the ALDH superfamily catalyze the removal of toxic aldehyde metabolites during stress-associated oxidative damage [61]. Differences in their thermal properties could shape cellular detoxification capacity under heat stress. These rankings are intended to guide experimental selection rather than establish mechanistic significance. Recombinant-protein melting assays, substrate- or cofactor-dependent thermal shifts, enzyme-activity measurements, and targeted mutagenesis will be required to determine the molecular basis of the observed differences.

Several limitations should be considered. Although the atlas contained 3917 proteins, it represents only part of the *Arabidopsis* proteome and is likely biased toward abundant and soluble proteins. Dataset size decreased further when structural, family, sequence, and functional information were jointly required; the family-adjusted structural model included 1615 proteins, and the paralog structural analysis encompassed approximately 1048 pairs. These reductions may limit the detection of small effects and family-specific patterns. In addition, the measurements were obtained under a single lysate condition and may differ with extraction buffer, ligand retention, protein concentration, or sample source. Furthermore, this uniform condition may not fully capture how intracellular environments modulate thermal stability in vivo, including differences between stress-induced and constitutive expression contexts; integrating transcriptomic data with thermal proteome measurements represents a promising direction for future research. Structural predictions and GO annotations also vary in completeness, particularly for poorly characterized plant proteins. Accordingly, the present results should be viewed as broad proteome-level trends that require validation in larger and independent datasets.

Overall, our findings suggest that gene-family identity provides the dominant framework for *Arabidopsis* protein thermal stability, whereas sequence evolution, protein architecture, and functional specialization contribute additional variation among paralogs. Most family members retain broadly similar thermal properties, but a subset displays divergence beyond that expected from sequence similarity. These exceptional differences do not appear to arise through a universal structural mechanism, but instead reflect heterogeneous, pair-specific molecular changes.

## 4. Materials and Methods

### 4.1. Data Sources and Study Design

This study was a secondary analysis of previously published *Arabidopsis* thermal-proteome data [10]. The original dataset was generated by thermal profiling of *Arabidopsis* protein lysates followed by quantitative mass spectrometry and melting-curve fitting. Details of plant growth, protein extraction, thermal treatment, mass spectrometric acquisition, protein identification, and melting-curve estimation are available in the original publication.

Protein identifiers were standardized to UniProt accessions. Duplicate records corresponding to the same protein were collapsed to obtain one protein-level melting temperature. Proteins lacking an unambiguous identifier or a valid melting temperature were excluded. After preprocessing, 3917 non-redundant *Arabidopsis* proteins were retained for subsequent analyses.

### 4.2. Evaluation Against an Independent Thermal-Proteome Dataset

The reproducibility of the lysate-derived melting temperatures was evaluated using *Arabidopsis* protein measurements from the Meltome Atlas (https://meltomeatlas.proteomics.wzw.tum.de/master_meltomeatlasapp/, accessed on 24 November 2025) [20]. The Meltome dataset was used only for independent data evaluation and was not included in any subsequent protein-family, paralog, structural, or functional analysis.

Protein identifiers from the two datasets were converted to UniProt accessions. Duplicate entries within each dataset were collapsed to obtain one melting temperature per protein. A total of 1572 proteins were shared between the two datasets. Agreement between the two sets of melting temperatures was evaluated using Pearson’s correlation coefficient. A fitted linear-regression line and the identity line were included in the corresponding visualization.

### 4.3. Protein-Family Annotation

Protein-family annotations were obtained by integrating UniProt and InterPro information. UniProt family annotations were used as the primary source. For proteins lacking an informative UniProt family assignment, InterPro family or conserved-domain annotations were used as supplementary classifications.

Family descriptions were standardized by removing redundant prefixes, harmonizing capitalization, and merging synonymous annotations. Of the 3917 proteins in the thermal dataset, 2954 were assigned using UniProt annotations, 576 were assigned using InterPro annotations, and 387 remained unannotated.

Unless otherwise stated, family-level analyses were restricted to protein families containing at least five proteins with valid melting temperatures.

### 4.4. Characterization of Family-Level Thermal Stability

For each protein family, the number of detected members, mean and median melting temperatures, within-family standard deviation, and mean pairwise absolute melting-temperature difference were calculated.

Mean pairwise thermal divergence was calculated as the average absolute difference in melting temperature across all unique protein pairs within a family. This measure was used to describe the overall thermal heterogeneity of each family.

The contribution of protein-family membership to proteome-wide thermal variation was evaluated using a random-intercept linear mixed-effects model, with protein family included as the random grouping factor. Between-family and residual variance components were extracted from the model, and the intraclass correlation coefficient was used to estimate the proportion of total thermal variation associated with family identity.

### 4.5. Randomization Analysis of Within-Family Thermal Similarity

To test whether proteins within the same family were more thermally similar than expected by chance, each family’s observed mean pairwise absolute T_m_ difference was compared with a size-matched null expectation generated by 1000 random sampling iterations without replacement from the full dataset. Observed and expected values were compared using a paired two-sided Wilcoxon signed-rank test. To account for family-size effects, thermal divergence was regressed against family size, and the resulting residuals were used to classify the lower and upper 10% of families as relatively conserved and diversified, respectively.

### 4.6. Construction of Non-Redundant Paralog Pairs

All possible protein pairs were generated within each annotated family. Self-pairs were removed, and protein identifiers within each pair were ordered consistently to eliminate reverse duplicates. Pairs were retained when both proteins had valid melting temperatures and available sequence-similarity information. The observed thermal difference for each pair was defined as the absolute difference between the melting temperatures of the two proteins.

Sequence-similarity values were standardized to a scale from 0 to 1. Values originally reported as percentages were divided by 100. The relationship between sequence similarity and pairwise melting-temperature difference was evaluated using Spearman’s rank correlation.

### 4.7. Sequence-Adjusted Thermal Divergence

Because closely related proteins are generally expected to exhibit more similar thermal properties, pairwise melting-temperature differences were modeled as a function of sequence similarity.

For each paralog pair, the sequence-adjusted thermal residual was calculated as the observed pairwise melting-temperature difference minus the value predicted from the fitted sequence-thermal stability relationship. Positive residuals therefore indicate greater thermal divergence than expected from sequence similarity, whereas negative residuals indicate greater thermal conservation than expected. Pairs in the upper 10% of the residual distribution were defined as exceptionally thermally divergent, and pairs in the lower 10% were defined as exceptionally thermally conserved. These classifications were based on the relative residual distribution and were not interpreted as formal tests of evolutionary selection.

### 4.8. Duplication-Mode Annotation

Gene-duplication modes were identified using DupGen_finder software (v1.0.0) [62]. The pipeline classifies duplicated gene pairs according to genomic position, collinearity, and sequence similarity into five categories: whole-genome or segmental duplication, tandem duplication, proximal duplication, transposed duplication, and dispersed duplication.

### 4.9. Structural Data Integration

Protein structures were matched to the thermal dataset using UniProt accessions and structural information from AlphaFold2 (https://alphafold.ebi.ac.uk; accessed on 24 November 2025). Structural data were available for 2611 of the 3917 proteins. A total of 2441 proteins had both structural and family annotations, and 1615 proteins with sufficient structural information and family representation were included in the final family-adjusted model.

The analyzed structural features included protein length, mean relative solvent accessibility, hydrophobic solvent-accessible surface-area fraction, hydrophobic-core score, packing density, α-helix fraction, β-sheet fraction, random-coil fraction, hydrogen-bond density, salt-bridge density, disulfide-bond density, π-π interaction density, and cation-π interaction density.

### 4.10. Proteome-Wide Associations Between Structure and Thermal Stability

Univariate relationships between individual structural features and protein melting temperature were evaluated using Spearman’s rank correlation. To compare proteins at the two extremes of the thermal-stability distribution, proteins in the lowest and highest melting-temperature quintiles were compared using two-sided Wilcoxon rank-sum tests. Resulting *p* values were corrected for multiple testing using the Benjamini–Hochberg method. Independent structural associations were subsequently evaluated using a linear mixed-effects model. Standardized structural features were included as fixed effects, and protein family was included as a random intercept to account for shared family ancestry.

### 4.11. Pairwise Structural-Divergence Analysis

For each paralog pair, the absolute difference in each standardized structural feature was calculated. A composite structural distance was then derived by integrating the available standardized structural-feature differences for each pair. Pairs with insufficient structural information were excluded. The final pairwise structural-divergence dataset contained 1048 non-redundant paralog pairs. The association between composite structural distance and sequence-adjusted thermal residual was assessed using Spearman’s rank correlation. Differences in individual structural features were evaluated separately and subsequently included together in a multivariable linear model. Duplication mode was included as an additional covariate.

### 4.12. Gene Ontology Annotation and Pairwise Functional Distance

Gene Ontology annotations were compiled separately for biological process, molecular function, and cellular component. Direct GO annotations were used for pairwise functional-distance calculations to avoid excessive inflation of similarity through propagated ancestral terms.

For each ontology, functional similarity was quantified using the Jaccard overlap between the GO-term sets of the two proteins. Functional distance was defined as one minus this overlap. A value near zero therefore indicates similar annotation profiles, whereas a value near one indicates little or no shared annotation.

### 4.13. GO Enrichment Analysis

Proteins represented in the lower and upper 10% of the thermal-residual distribution were collected as exceptionally conserved and exceptionally divergent gene sets, respectively. Repeated protein identifiers within each group were removed. GO enrichment analysis was performed separately for biological process, molecular function, and cellular component using clusterProfiler (v4.18.4; Bioconductor, Boston, MA, USA) and the corresponding *Arabidopsis* annotation database. All genes represented in the analyzed paralog-pair dataset were used as the background universe. Enrichment significance was evaluated using a hypergeometric test, followed by Benjamini–Hochberg correction. The most strongly enriched terms in each ontology and residual group were selected according to adjusted *p* value and gene ratio.

### 4.14. Broad Subcellular-Localization Analysis

Broad subcellular-localization categories were inferred from GO cellular-component annotations. Propagated cellular-component annotations were included in this analysis to improve the assignment of proteins to major compartments.

A paralog pair was classified as having overlapping localization when the two proteins shared at least one broad localization category. Pairs without a shared category were classified as non-overlapping.

### 4.15. Integrated Prioritization of Candidate Paralog Pairs

Candidate paralog pairs were ranked using a composite score integrating sequence-adjusted thermal residual, observed pairwise ΔT_m_, overall GO annotation distance, disulfide-bond-density difference, and melting-curve quality. Each metric was converted to a percentile rank and weighted by 40%, 25%, 15%, 10%, and 10%, respectively. Pair quality was defined by the lower R^2^ value of the two melting curves. When a component was missing, the score was renormalized using the available weights.

### 4.16. Statistical Analysis and Visualization

All data processing and statistical analyses were performed in R (v4.5.2; R Foundation for Statistical Computing, Vienna, Austria). Statistical tests were two-sided unless otherwise specified. Pearson’s correlation was used for comparison between the independent thermal datasets, whereas Spearman’s rank correlation was used for monotonic associations involving sequence similarity and structural variables. Wilcoxon tests and Kruskal–Wallis tests were used for non-parametric comparisons.

Multiple-testing correction was performed using the Benjamini–Hochberg method. An adjusted *q* < 0.05 was considered statistically significant. Effect estimates are reported with 95% confidence intervals.

## Data Availability

The *Arabidopsis* lysate thermal-profiling dataset was obtained from Lyu et al. [10]. The *Arabidopsis* Meltome dataset used for independent evaluation was obtained from Jarzab et al. [20]. Duplication-mode annotation was performed using DupGen_finder v1.0.0 [62].

## Supplementary Materials

The following supporting information can be downloaded at https://www.mdpi.com/article/10.3390/plants15172617/s1, Table S1: Protein thermal stability data in this study; Table S2: Thermal divergence and conservation status of Arabidopsis protein families; Table S3: Protein-family annotation, duplication mode, sequence similarity, and sequence-adjusted thermal divergence of Arabidopsis paralog pairs; Table S4: Melting temperatures, protein-family annotations, and standardized structural features of Arabidopsis proteins; Table S5: Sequence similarity, duplication mode, thermal divergence, and pairwise structural differences of Arabidopsis paralog pairs; Table S6: Effect estimates and multiple-testing-adjusted associations of structural-feature differences with sequence-adjusted thermal divergence among Arabidopsis paralog pairs; Table S7: Gene Ontology enrichment statistics for proteins from exceptionally thermally conserved and divergent Arabidopsis paralog pairs.
